# Domain Specificity vs. Domain Generality: The Case of Faces and Words

**DOI:** 10.3390/vision8010001

**Published:** 2023-12-21

**Authors:** Paulo Ventura, Francisco Cruz

**Affiliations:** Faculdade de Psicologia, Universidade de Lisboa, 1649-003 Lisbon, Portugal; franciscocorreiadacruz@gmail.com

**Keywords:** domain generality, domain specificity, word processing, face processing, holistic processing

## Abstract

Faces and words are ever-present stimuli in social environments that require fine-grained, efficient discrimination of their constituents in order to acquire meaning. Provided that these stimuli share multiple characteristics, while simultaneously being different visual object categories in important ways, a debate has ensued pertaining to whether their processing can be reduced to a common mechanism or whether each category mobilizes exclusive resources. We thus first present briefly domain-specific and domain-general accounts, as opposing perspectives that highlight the absence and presence of commonalities in face and word processing, respectively. We then focus on how faces and words are processed. While faces are usually associated with holistic processing of facial features, to create a perceptual whole, there is no such consensus pertaining to word processing. Words have been argued to rely on either letter-by-letter processing or in a way closer to that of faces, since they are also objects of expertise. Lastly, we advance the debate by providing an overview of our latest research findings. These findings provide a more direct comparison of face and word processing, by incorporating both stimuli in one task concurrently.

## 1. Domain Specificity vs. Domain Generality: The Case of Faces and Words

Faces are ever-present stimuli in social environments, requiring fast between-category (i.e., of a face as such) and within-category (i.e., identity of a face) discrimination. Similarly, given the importance of communication in these environments, words are another ubiquitous stimulus category, and extraction of their meaning is also contingent on within-category judgments (i.e., parsing the identity of a letter sequence). Provided that both faces and words are frequent objects that share several properties, there is a debate on the extent to which these rely on shared or distinct cognitive processes and resources (see [1,2,3]). According to domain-specific accounts, faces and words are distinct object categories that rely on largely independent, modular mechanisms [4]. Alternatively, domain-general accounts claim that there is a shared pool of resources that both face processing and word processing resort to, potentially leading to resource competition [5].

In the present paper, we seek to explore both domain-specific and domain-general accounts in the context of faces and words. We first explore the notion of domain specificity and domain generality in the context of visual object processing. Then, we detail the findings of the literature on how faces and words are processed, namely the role of holistic processing in face and word recognition. Specifically, we focus on the composite task as a paradigm designed to tap into this type of object processing. We do so provided that others have argued for expertise-based accounts of word processing. These suggest that words rely on processes similar to those deployed when in the presence of face stimuli—that is, holistic processing (see [4,6,7,8,9,10,11]). In doing so, we focus on both the classical paradigm and a novel task that taps into holistic processing for faces and words simultaneously. Using the latter, in which face and word processing are pitted against one another concurrently, we more directly compare the extent to which faces and words resort to shared resources and processes. We detail results from our own research findings, in which we systematically compare the holistic processing for different visual object categories (i.e., faces, words, and Gestalt lines). We detail how this approach can inform the debate on domain generality vs. domain specificity for faces and words, as well as identify the locus of the holistic processing for these stimuli.

## 2. Domain-Specific vs. Domain-General Perspectives

The domain-specific versus domain-general debate is one about the extent to which specialized structures address specific cognitive tasks, as opposed to general mechanisms that subserve multiple cognitive operations. It is a debate that extends over a wide range of human cognition domains, such as theory of mind [12], creativity [13,14], reasoning and overconfidence [15,16], language [17,18], mathematics [19], music perception [20], and working memory [21].

### 2.1. Domain Specificity

To say that a mental operation is domain-specific is, essentially, to argue that the structures enabling its functioning are narrow. Originally, Fodor [22] put forward the notion of modules. Modules describe structures that (1) process a limited set of stimuli (being unresponsive to inputs beyond their scope), and (2) do so in an essentially fast and automatic fashion (see also [23]). Domain specificity has been espoused by functional perspectives of the mind [24]. The environment poses specific, well-defined challenges to individuals, which are better managed by systems designed to address them. Thus, evolution presses towards the creation of task-bound modules, tailored to solving specific and discrete problems.

Given that faces have always been common stimuli in social environments, the development of a tailored module for processing them is not theoretically counterintuitive. Reading acquisition, however, is a recent activity in the evolutionary timescale—making the possible existence of a word processing module more puzzling [4]. Barrett and Kurzban [25] argue that such a module could be acquired by further specialization of a face processing module into two, attuned to each of these visual categories. As modules are designed towards specific stimuli properties, this would require that words and faces are similar in meaningful ways, while being distinct enough to justify the bifurcation. Indeed, the visual properties of faces and words differ substantially, justifying the sub-specialization of an already existing module. For example, face perception is tuned to objects whose features are not isolated and are reliant on depth processing, but words are two-dimensional objects, made of parsed two-dimensional components (i.e., letters) with a different configuration [26].

If there are specialized—and independent—modules designed to process faces and words, it follows that, when one is damaged, deficits in performance should only emerge for the category in which the affected module specialized. In line with this, subjects with developmental prosopagnosia (DP)—a persistent deficit in face recognition—have been shown to retain most capabilities associated with object processing in general ([27,28,29]; also seen in acquired prosopagnosia [30]), and word processing, specifically (cf. [1]). The performance in reading longer words or in lexical decision tasks (i.e., discerning words from non-words) does not differ between healthy controls and DP participants [31,32]. Similarly, DP participants do not differ from healthy controls in letter and word naming times, and also display a naming advantage for letters included in words (i.e., the word superiority effect [33]). These deficits in face recognition with intact word recognition have been found even when the impairment was acquired, rather than developmental [34]. The reverse has also been shown to hold true: both individuals with acquired and developmental forms of dyslexia (DD)—a generalized difficulty in word recognition—display intact face recognition capabilities [30]. While DD participants perform worse than healthy controls in lexical decision tasks, no differences in performance emerge in face memory tests [35]. These results extend to activation patterns, such that DD participants and healthy controls do not differ in their event-related potentials (ERPs) recorded during a face memory task [36].

The modular view argues that these dissociable cognitive operations should be located in discrete, independent underlying brain regions. Many authors have argued for discrete brain regions dedicated to processing specific object categories, such as places, bodies, faces, and words (e.g., [37,38]). Kanwisher and collaborators [39] found a region in the right fusiform gyrus that is particularly sensitive (i.e., more strongly activated) to faces (vs. objects)—the “fusiform face area” (see also [40]). Similarly, a corresponding region for words has been found in left inferior temporal regions. This area—the “visual word form area” (VWFA)—displays a higher activation for written words than for consonant strings ([41]; see also [42,43,44,45]). The opposite lateralization for faces and words has been argued to support domain specificity for these object categories [46,47]. Opposite lateralization for faces and words has been framed to reflect the existence of independent and selective regions designed to process faces and words (i.e., in the right and left hemisphere, specifically; henceforth RH and LH). The development of LHG lateralization for words and RH lateralization for faces may depend on spatial frequency content [48]. Thus, the left lateralization for reading may be an outgrowth of an evolutionarily older specialization of the LH at the basic sensory level, namely the ability to detect fine edges and sudden changes in visual space, referred to as high spatial frequency (HSF) visual information. The specialization of the RH for faces may be related to a dependency of faces on low spatial frequencies. However, Ossowski and Behrmann [49] suggest that, rather than serving as a precursor for the LH superiority for word recognition, the LH bias for HSF input might emerge in concert with it or potentially even be a consequence of the acquisition of orthographic competence.

### 2.2. Domain Generality

While modules have been thought of as “being directed at” specific input categories (faces or words), domain-general explanations posit that cognitive operations instead act on inputs provided by multiple categories instead, such as faces and words [23]. Domain-general accounts highlight structural and functional interdependence [50]. Due to commonalities in the stimuli being processed, one pool of cognitive resources, structural networks, and/or computations is recruited when either of them is present.

According to the many-to-many hypothesis, not only are face and word processing reliant on multiple regions, but there is also overlap between them, such that many contribute to the processing of both visual categories [26,51,52,53,54]. Rather than being governed by discrete modules, face and word recognition rely on shared bilateral networks, although each hemisphere’s contribution is different for these two categories. These shared networks prompt competition, especially because within-category homogeneity is high in both faces and words. To increase efficiency, and considering that language processing is often left lateralized, this region becomes more attuned to the orthographic dimension of word processing. Consequently, and since faces and words cannot fully overlap in their processing structures, face representations gradually become more right-lateralized. Put differently, the earlier language-biased left hemisphere lateralization for words drives a later right hemisphere lateralization for faces. In any case, though there is face and word dominance in the right and left hemisphere, respectively, both processes remain bilateral, with efficiency being predicted by the extent to which both hemispheres are involved.

This proposal runs counter to the idea of clear-cut dissociations, suggesting that deficits in both face and word processing should ensue in individuals with either DP or DD. In line with this, impairments in processing either faces or words are paired with increased difficulties in processing the other category. Research with higher powered samples finds that the severity of face recognition impairment in prosopagnosia predicts slower reading speeds in both developmental and acquired deficits [1]. Subjects with prosopagnosia additionally display a stronger word length effect (i.e., there is an increase in reading time as words become longer [55]), as well as deficits in capturing other word properties (e.g., style–font vs. handwriting [55,56]). Conversely, dyslexic participants’ reading difficulties scores were better predicted by a decreased performance in matching face stimuli (vs. other visual stimuli [5]). Abnormal word feature processing has been found, as expected, in individuals with DD, but this feature processing impairment extends to facial features in this population [57]. Additionally, this population has been found to display abnormal lateralization for faces and words (i.e., there was no lateralization, as found in healthy individuals [50]).

Another argument for domain generality across faces and words pertains to the existence of general performance factors. If multiple operations are reliant on general processing mechanisms, a general factor of performance should account for the performance across these operations. Indeed, the majority of variance in recognition of novel object categories can be reduced to such a factor, hinting at the presence of a common system dedicated to processing of novel visual stimuli, regardless of their category [58]. Maratos and collaborators [59] extended these conclusions to known objects, by finding an underlying component that reflects performance for bodies, cars, faces, and words. This general recognition factor is also found in super recognizers (i.e., with superior face recognition abilities), who are better than controls in recognizing other visual objects, such as words [60].

Faces and words are good candidates in the context of domain generality, as these have many commonalities. Both are stimuli categories with whom people are familiar, and therefore, experts on [4,8,9,10,11]. Furthermore, both are described as a global structure that relies on fine-grained discriminations (i.e., of features or letters) to acquire meaning (i.e., in identity judgments and word identification). In the many-to-many hypothesis, lateralization is reframed as a development compatible with domain generality and geared towards increasing the processing efficiency of faces and words. The existence of a fully shared system for processing both faces and words would lead to conflict, due to competition to access a shared pool of limited resources. In any case, incomplete lateralization, such as that found for faces and words (i.e., both gyri recruited when presented either category) allows for increased efficiency [26,61,62].

Barrett and Kurzban [25] argue that evolutionarily novel activities, such as reading, can theoretically be allocated to previously created structures designed to address similar cognitive tasks. In the context of faces and words, this suggests that, during literacy acquisition, the ensuing conflict would push for the right lateralization of face processing [53,63,64,65]. This suggests that hemispheric lateralization for faces is brought about by with the acquisition of word processing. As the former is “pushed” by the acquisition of the later, abnormal lateralization should mostly be found for faces alone (or for both faces and words). In line with this, Collins and collaborators [66] found that DP participants only display abnormal ERP responses in the right hemisphere, suggesting abnormal lateralization for faces. No evidence of abnormal lateralization was found in participants with DD. Additionally, Furubacke and collaborators [67] showed that the hemispheres are complementary. In their task, participants were presented with face and word stimuli that were interleaved and were asked to provide same–different judgments based on specific stimuli properties. When the property of the stimulus and that of the filler (interleaved) one were traced to the opposite hemispheres (e.g., face and word identity to the right and left hemispheres, respectively), there was no interference. However, when the intended property and filler property (of the interleaved stimulus; e.g., face and handwriting identification) were lateralized to the same hemisphere (e.g., right hemisphere [68]), interference effects were found. These experiments support domain-general accounts in face and word processing as a whole and support specific predictions of the many-to-many hypothesis (outlined above), aligning with the predictions of the many-to-many hypothesis regarding lateralization.

## 3. How Are Faces and Words Processed?

Though the debate between domain-specific and domain-general accounts for the processing of faces and words often focuses on dissociations and brain activation, there is a parallel question that further helps address the extent of their interdependence—specifically about how faces and words are processed. Whether faces and words rely on similar or distinct processes is orthogonal to where such operations take place. Similar processes can be computed by distinct structures; conversely, one same structure can be involved in multiple cognitive processes. In any case, exploring the extent to which the computations involved in processing a face match those enforced when presented words can further elucidate the debate on whether these are domain-general or domain-specific.

Holistic processing has been regarded as one of the mechanisms underpinning the ability of the visual system to fulfill the task demand of fast individuation of faces [69,70,71,72,73,74]. Face identification requires that one goes beyond the mere recognition of a face as such, considering minute variations in its constituents (e.g., distance between eyes and nose) in order to match a face with their corresponding identity. This requires a highly effective processing system, which operates under principles that maximize the extraction of these fine-grained distinctions—and such rapid individuation is attained via holistic processing [69,70,71,72,73,74]. Holistic processing has had different definitions in the literature. Two of the most studied definitions involve a perceptual strategy of processing all parts together that becomes automatized with experience and/or due to a history of learned attention to diagnostic parts [6,10,11], and the explicit representation of spatial relationships between features (e.g., [69,75]). Rather than undifferentiated wholes, face recognition involves representations of both the local elements (individual face parts) and their configuration (e.g., [74,76,77,78]). Holistic processing can thus be defined as the obligatory encoding of/attending to all object parts, which in turn are also encoded and represented independently [79]. Central to acquiring such processing capabilities is the extensive contact with such category of stimuli that prompts expertise in subsequent judgments [6,7,10,11].

The holistic processing of faces has been commonly assessed through three different tasks: the inversion task, the part-whole task, and the composite task (which will be explored in the next section). The inversion task ([80]; see also [81,82,83,84]) assumes that holistic processing should be attuned to face configurations that are aligned with those people are usually exposed to, or to upright configurations. Though inverted faces retain the features of a regular face, their relative positioning is reversed, thus potentially disrupting the processes that would otherwise be activated. On the one hand, identifying upright faces should be easier than identifying inverted faces, because only the former activates holistic processing, which is highly efficient. On the other hand, the decrease in performance for inverted faces should be higher than for other objects that do not rely on holistic processing [85]. Both predictions for faces find support in the literature [83], thus suggesting that the configuration of (upright) faces activates a specific processing mode—holistic processing—that is beneficial for stimuli recognition.

The part-whole task ([78]; see also [86]) assumes that holistic processing drives resources towards an integrated whole, and away from individual face features. This should benefit the identification of a face but hinder the recognition of specific face features. In this task, participants are usually presented with a target face and then two response options to choose from, their goal being to identify which of them matches the previously presented face. In some trials, the response options are whole faces (whole trial), whereas in others the decision is between specific face features (e.g., nose; part trial). Participants’ performance is better in whole trials, relative to part trials, thus suggesting that faces are processed as integrated wholes, rather than as a sum of their features. Such an effect is not found when holistic processing is not present (e.g., in houses [78]), suggesting that indeed the advantage for (regular) faces pertains to the involvement of holistic processing.

Word processing has been argued to rely on the extraction of information from word features, or part-based processing. Characteristics contingent on attending to the entirety of a word (e.g., number of letters) are not enough to reveal word identity (as there are several words with each given number of letters [87]). Therefore, configural processing alone would be insufficient in proper word processing. In fact, faces are commonly found to recruit more holistic processing than other visual objects, suggesting that face processing is a category particularly tuned to configural processing [76]. Therefore, Farah [76,88,89] proposed a continuum ranging between part-based and whole-based (holistic) processing, associated with words and faces, respectively, with other object categories in between. In line with this, participants with alexia (acquired difficulty in reading) have impairments in representing objects’ parts, hence the deficits in their word recognition [90]. Computational models of reading tuned to letter-by-letter processing perform similarly to humans (i.e., taking longer to identify longer words) [91]. This suggests that attending to individual word features (i.e., letters)—in a part-based fashion—is central to adequately identifying word identity.

Some authors, however, argue that words are processed holistically. Reading acquisition is essentially about acquiring expertise in fast identification of letter strings comprising similar elements [92,93]. Experts in multiple object categories, such as birds [94], cars [95], and musical notation [96], for example, have been shown to process these objects holistically. In this context, holistic processing, more than being specific to faces, has been described as a marker of expertise in a given visual category [79]. Provided that word identification requires high specialization, as well as the high degree of exposure to words, words have been paired with faces as objects of expertise [4,8,9,10,11]. In recent years, the perceptual expertise framework for word processing has harnessed substantial attention (e.g., [97,98,99]), partly due to the specifics of word identification. Efficient visual word recognition requires fast identification of letters and of their position within the word, many of which only hold minor perceptual differences (e.g., <G> only differs from <C> in one minute horizontal segment; <GOD> and <DOG> are composed by the same letters, but in different order). Furthermore, low-level features (e.g., case; <god> and <GOD>) change across multiple visual representations of one same abstract orthographic representation (for a recent review, see, [100]).

**Figure 1:**
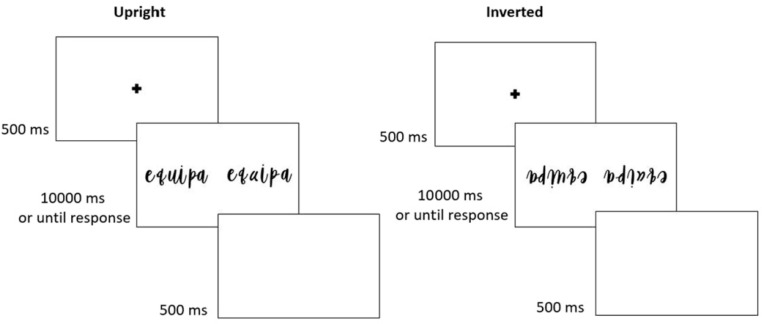
The configural sensitivity task with Portuguese words. Two versions of a word with different spatial relationships between letters, as used in a different trial. Sequence of events in an upright trial and an inverted trial. Adopted from [103], Attention, Perception, & Psychophysics, 2022, 84, pp. 1737. SpringerNature.

**Figure 2:**
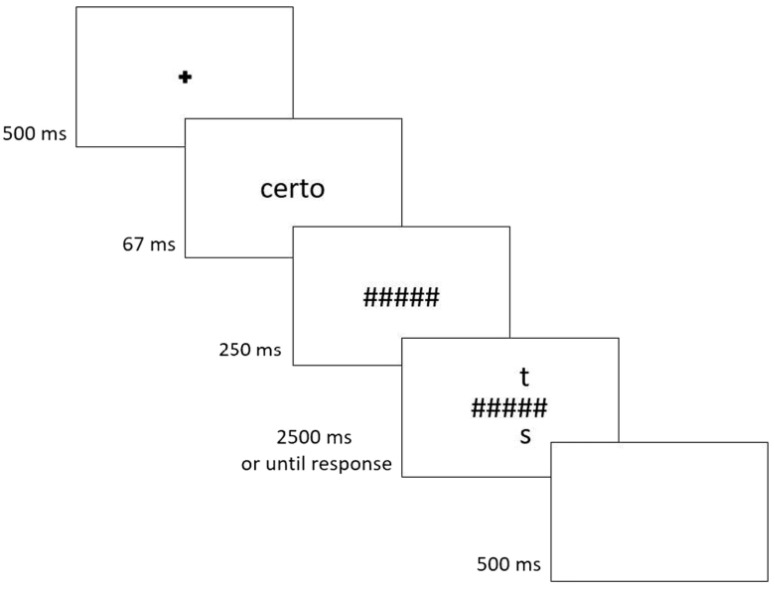
The part-whole task with Portuguese words. Adopted from [103], Attention, Perception, & Psychophysics, 2022, 84, pp. 1737. SpringerNature.

Under an expertise framework, effects previously found when studying the holistic processing of faces should generalize to words, since it is also a category of expertise (For the effects of the present literature review, we are specifically interested in this claim. Note, however, that, beyond the expertise framework of visual expertise, several distinct tasks have been used to evaluate face and word processing. Many of these do not transfer from one domain to the other, due to pertaining to specific properties of the stimuli. For a battery of tasks used in the face and word processing literature, see Robotham et al. [101]). Indeed, the expressions of holistic processing previously found for faces, i.e., the inversion (e.g., [80,102]), the part-whole (e.g., [78]), and the composite (e.g., [74]) effects, have also been reported in visual word recognition (see [103]). Recognition of upright words, for example, is superior to that of inverted words [104]. More importantly, changes that disrupt the holistic configuration of a word hinder performance more for upright (vs. inverted) stimuli, and more so than changes that did not disrupt the configuration of the word [105,106] (Figure 1). Additionally, letters are more easily recognized when integrated into a word (vs. in isolation)—an effect termed the word superiority effect [107,108] (Figure 2). This is similar to the main finding reported by authors using the part-whole task with faces (e.g., [78,86]), and has been argued to reflect the conjoint integration of single letter processing with top-down information arising from whole-word lexical representations (see [109,110]). Overall, evidence for the holistic processing of words is robust, especially considering that it has been found across scripts with different orthographic and phonetic characteristics (English: [93]; Portuguese: [111]; Chinese: [112]).

If there is holistic processing of words that reflects expertise in the object category, then the more of an expert one is in word stimuli, the more holistic processing should be involved. Indeed, participants with more reading experience engage more in holistic processing of words [93]. Conversely, the deficits found in readers with dyslexia are attenuated by the extent to which their word processing is holistic [113]. Finally, inversion effects have been found to be stronger for controls, relative to dyslexic individuals (i.e., with less expertise), showing that expert readers are more reliant on holistic processing to extract the meanings of word stimuli [105].

In any case, whether words elicit contrasting holistic and part-based processing might constitute a false dichotomy. Rather than an all-or-nothing phenomenon, holistic and part-based processing might be two ends of a continuum, and complex stimuli categories might recruit processes with varying degrees of holistic processing. Face recognition is not purely holistic, including representations of its parts, conjoint with the whole face representation [74,76,77]. Similarly, though many features extracted through holistic processing (e.g., number of letters [87]) are not enough for word identification, holistic processing is still required for fast and accurate word identification [93]. Ventura and collaborators [114] found that behavioral responses to faces and words are different. While detecting a change in a face (which benefits from holistic processing) was easier than identifying its location (hindered by holistic processing, but benefitting from feature processing), change detection and localization was similar for words. In control, non-expertise stimuli, it was easier to identify the location of a change than whether it had taken place. These results suggest that objects of expertise found to benefit from holistic processing—i.e., faces and words—may rely on other processing strategies (e.g., part-based) to different extents. Despite the possibility of some noise in encoding positional information (e.g., bigrams; [115,116]) being able to identify specific letters and their positions is crucial in orthographic processing, thus requiring the recruitment of part-based processes—making the mechanisms subserving face and word processing not fully overlapping.

## 4. Domain Generality Revisited: The Composite Task and Holistic Word Processing

Holistic processing is the automatic integration of individual elements of a stimulus into a whole, even though the parts may be concurrently represented independently [79]. From this definition, it follows that judgments on a portion of a face should be influenced by the remainder of the information included in the representation. The composite task rests on this rationale (cf. [72,79]). In this task, participants are presented two faces in sequence, and are asked to identify whether one face half (e.g., upper half) is the same in the two stimuli. Holistic processing is supported through the influence that the irrelevant part (e.g., bottom half) has on judgments of the target half. When the irrelevant half of the face is congruent (i.e., prompts the same response as the target half) the performance is better than when the irrelevant half is incongruent. This effect, however, is hindered when the two halves (upper half and lower half) are not aligned—in which case, people can process the target half independently, instead of as an integrated whole.

**Figure 3:**
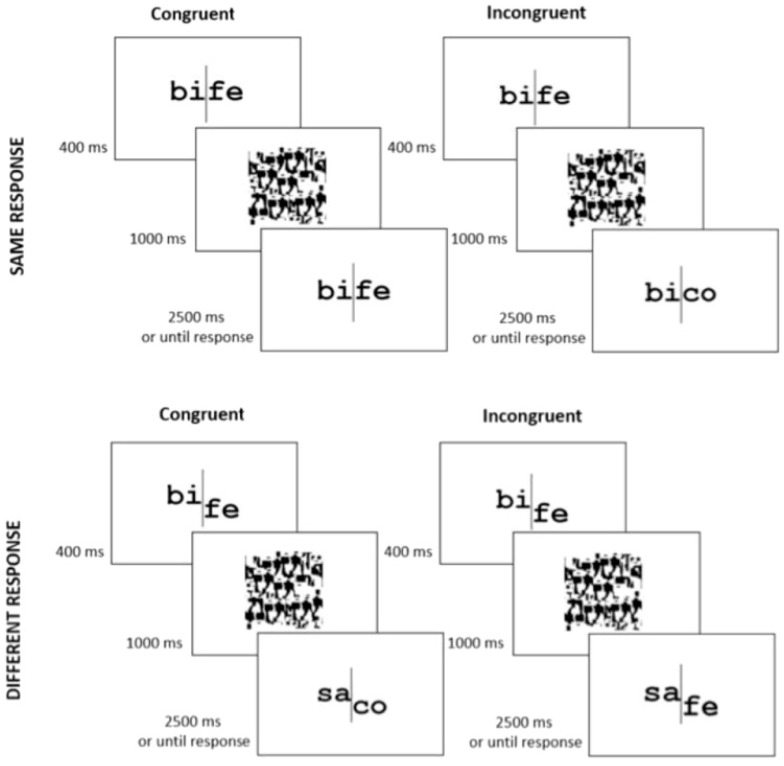
The composite task with Portuguese words. In this example, participants should judge if the left half of two sequentially presented words are the same or different. An example trial is shown for each of the congruency × response conditions. Adopted from [103], Attention, Perception, & Psychophysics, 2022, 84, pp. 1736. SpringerNature.

This paradigm has been adapted to test the holistic processing of words [93,111,112,117] (Figure 3). In the context of words, two syllable words are presented in sequence and participants must attend to one of the syllables, in a same–different judgment. This is a perceptual task where reading is not required: participants are asked to perform a same–different matching task on a specific visual part (e.g., the first syllable) of two sequential (dissyllabic) words and not on whole strings (e.g., same-response trials: BIFE–BIFE; different-response trials: BIFE–SAFE). Two critical components in this task argue for the holistic processing of words. First, the influence of the irrelevant part (e.g., the right half) on performance, over the target part (e.g., the left half); that is, a significant congruency effect: there is better performance when the irrelevant part is congruent in response to the one induced by the critical part (in same-response trials: e.g., BIFE–BIFE, as the critical and irrelevant parts are the same; in different-response trials: e.g., BIFE–SACO, as both the critical and irrelevant parts induce a different-response) than when incongruent (in same-response trials: e.g., BIFE–BICO, because the critical part of the two words is the same but the irrelevant part is different; in different-response trials: BIFE–SAFE, as the critical part of the words is different but the irrelevant part is the same). Second, the congruency effect is modulated by alignment; that is, it is severely reduced when the two parts of the word are misaligned (e.g., the right part is moved down relative to the left part) rather than aligned, probably because the whole perception is disrupted.

Expert reading, which has been characterized as unfolding in a fast, parallel fashion, has been linked to this holistic processing, reinforcing the idea that global representations are a hallmark of expertise [103,118,119]. The holistic processing of words arguably emerges in later stages of processing, as it is unaffected by changes in stimuli properties extracted in earlier stages. Manipulations of surface word characteristics, such as font, do not interfere with holistic word processing [111]. Additionally, manipulating the type of visual information deleted from words (e.g., mid-segments vs. vertices) required for early letter identification did not modulate the holistic processing of words [120]. Such findings argue for holistic processing, as they are bound to late lexical elements of their representation. Conversely, word properties beyond surface level characteristics have been shown to modulate holistic processing. The composite word effect emerges for phonetically consistent (i.e., with consistent grapheme-to-phoneme mapping), but not phonetically inconsistent words [121]. Overall, these studies argue for a late locus to holistic word processing.

Regardless, there is evidence that highlights the role of earlier holistic word processing. Chen et al. [117] examined how early holistic processing of Chinese characters emerges, by recording ERPs in an adaptation paradigm. Participants judged if the top parts of two sequentially presented characters were the same or different, while ignoring the bottom part. The early potential (P1) at the posterior channels was smaller when the attended top parts were the same, compared with when they were different, indicating an adaptation effect. Critically, for trials with identical top parts, P1 was larger when the irrelevant bottom parts were different, indicating a release of adaptation. This effect was present only when the two-character parts were aligned but not misaligned, and only for characters but not for pseudo characters. The finding of early sensitivity to all parts of a Chinese character suggests that Chinese characters are represented holistically at a perceptual level.

The earliest marker of holistic processing for words, P1, thus seems to emerge earlier than the one associated with face processing (for words: [117]; e.g., for faces, N170: [122]). Evidence of holistic processing has been found for face presentation durations as short as 50 ms [9]. For words, no changes in holistic processing were found when longer presentation times were used (i.e., between 170 m and 600 m [123]). One should note, however, that there is an alternative explanation for these findings, as the P1 marker found in Chen and collaborators [117] has been found to be larger for attended (vs. unattended) stimuli, and thus believed to reflect attention modulation towards visual objects (e.g., [124]).

The early nature of holistic processing is not contingent to specificities of face or word stimuli, however. When using line patterns associated with salient Gestalt properties (e.g., closure), similar interference effects emerge [125]. This suggests two interdependent routes for holistic processing, one anchored on visual, early extracted stimulus information, essentially bottom-up, and another associated with abstract representations acquired through experience, mobilized in a top-down fashion [125]. This dual-account model may extend to the holistic processing of words, considering the evidence in favor of both an early [9,117] and a late [111,120,121] locus for holistic word processing. Indeed, the existence of both early and late loci for holistic word processing are supported by the functional organization of the VWFA. The VWFA is organized in an anterior-to-posterior fashion, with some regions focused on early stages of word processing, and others attuned to its later stages [126,127].

While the above-explored argues for the existence of holistic processing of words, whether this process is the same as that found in faces is still an open question. The existence of holistic processing for words and faces does not imply one same process, making use of the same pool of resources or recruiting the same brain regions. Therefore, a more complex paradigm should be considered when exploring whether there might be domain generality for both faces and words. Such a paradigm would be required to directly compare the processing of two object categories in the presence of one another, by creating competition among them. The interference in the processing of one visual object in the presence of another could be an informative proxy to understanding the extent to which the former is reliant on resources that are also mobilized by the latter.

Curby and Moerel [128] developed a paradigm with such characteristics, by presenting faces superimposed on Gestalt stimuli. As these stimuli incur in holistic processing, the superimposition creates a trade-off in holistic processing, such that when one processes the unattended category holistically, the attended category might be influenced. In line with this, the authors found that both faces and Gestalt line patterns were processed less holistically when the other category was presented in a way that promoted holistic processing (i.e., aligned vs. misaligned). Furthermore, this again suggests an early locus for the holistic processing of faces, since Gestalt line stimuli processing relies on lower-level visual properties that are extracted quickly.

This paradigm can be adapted to test whether there is holistic word processing, while answering some questions on the nature of such a process; namely, whether holistic word processing is modulated by the holistic processing of other object categories. If the holistic processing of different object categories is interdependent, this would speak to words’ domain generality. Conversely, if no trade-off in the holistic processing of words and other stimuli exists, the former can be deemed as domain-specific. As noted above, there is evidence arguing for early holistic processing in words, and lower level word properties, salient in Gestalt-like shapes (e.g., closure [129,130]), are pivotal for letter identity extraction and subsequent word identification. This being the case, there could be reciprocal influence between words and Gestalt stimuli. When superimposing words on Gestalt line patterns and vice versa (i.e., using Curby and Moerel’s paradigm [128]), Ventura and collaborators [131] found that interference in holistic processing was not reciprocal. Though aligned (irrelevant) words reduced the holistic processing of Gestalt line stimuli more than misaligned (irrelevant) words, superimposition did not lead to reduced holistic word processing. Thus, words are subject to early holistic processing, but resistant to some interference effects. This is probably due to their automatic processing and contingent extraction of lexical information, which is absent in Gestalt line stimuli.

**Figure 4:**
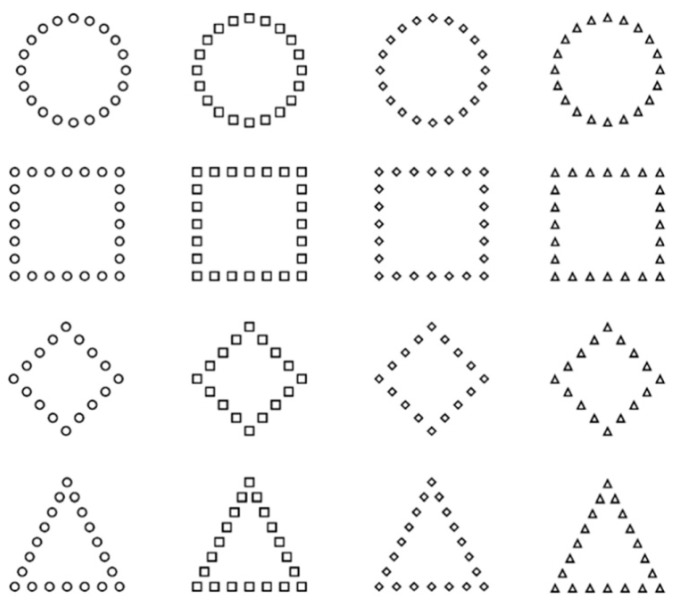
Compound hierarchical figures stimuli. Adopted from [134], Attention, Perception, & Psychophysics, 2021, 83, pp. 2193. SpringerNature.

**Figure 5:**
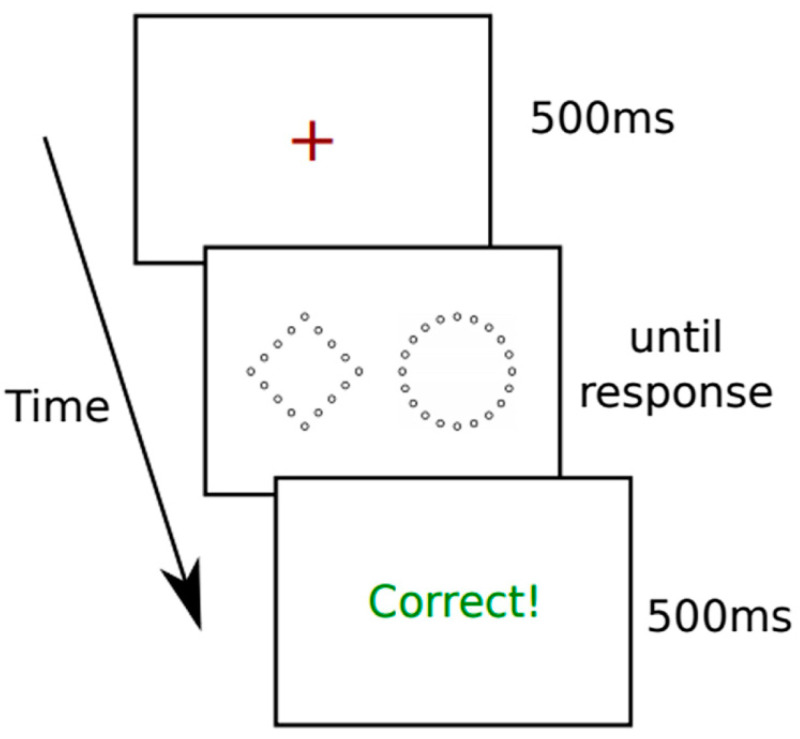
Stimulus sequence of experiment with hierarchical stimuli. Adopted from [134], Attention, Perception, & Psychophysics, 2021, 83, pp. 2193. SpringerNature.

Though both faces and words are associated with early holistic processing, it is not yet obvious that both stimuli categories are domain-general—in this context, that their holistic processing is one and the same. The Navon task (see [132,133] (Figure 4 and Figure 5)), in which participants are presented a figure whose pattern matches that of a letter/figure, comprising multiple smaller letters/figures, and have to match this target with another, by focusing either on the global (i.e., letter/figure) or local (i.e., smaller letters/figures), can prime processing approaches to be more or less holistic, respectively.

After performing a Navon task, participants display more or less holistic processing in a subsequent composite task, depending on whether they were prompted with global or local processing—and this holds for both faces and words as target categories in the composite task [134,135]. Additionally, though some authors have argued for opposite hemispheric lateralization in faces and words [39,41], Ventura and collaborators [136] found that, when word presentation was lateralized, holistic processing was primarily anchored to the right hemisphere, the one commonly considered as specializing in faces. These findings detail commonalities in face and word processing, while highlighting that both stimuli are processed holistically, hinting at a possible interdependence between these object categories.

Directly testing this interdependence can be achieved through the adaptation of Curby and Moerel’s [128] paradigm. When words are superimposed on faces, the former interfere with the holistic processing of the latter, leading to its reduction, and such interference is found mainly when words stimuli are aligned (vs. misaligned). Similarly, irrelevant aligned faces reduce holistic word processing, relative to misaligned face stimuli [137]). This reciprocal interference suggests a trade-off in holistic processing between faces and words; put differently, the processing of faces and words is not independent. Independence in object category processing, either in cognitive resources and/or brain regions recruited, is a fundamental property of modular accounts of the mind [31,32,33,138,139]—and thus this finding suggests that faces and words are domain-general, to some extent.

Despite the substantial overlap between face and word processing, these categories are not without their differences. As discussed in the section ‘How are faces and words processed?’, words may fall somewhere in between the ends of the continuum: holistic processing for faces, part-based processing for, e.g., houses. Taking these findings into account, a dichotomous view of holistic vs. part-based processing is rendered implausible, and continuum-centered proposals, such as Farah’s [76,88,89], emerge as more conciliatory. In any case, the findings presented above simultaneously argue for a revision of Farah’s [76,88,89] proposal. The results presented would place words in the middle of the continuum, combining holistic and part-based processing, while Farah’s [76,88,89] proposal anchors words in part-based processing instead. Face and word processing appear to be domain-general, but are not fully overlapping, such that the relevance of letter positioning in word identity requires parallel, part-based processes.

## 5. Conclusions

Faces and words are ever-present stimuli and, therefore, are visual objects on which people are experts. Overall, our evidence argues for substantial overlap between face and word processing, not only in terms of cognitive resources recruited, but also of neuronal structures mobilized in processing them—in line with the idea of domain generality. Faces are processed holistically, and so are largely words. In any case, there are important differences between faces and words that should be carefully considered.

## Data Availability

Not applicable.
